# Additional Role of Transabdominal Ultrasonography on Esophagogastroduodenoscopy for Uninvestigated Dyspepsia: A Prospective Study

**DOI:** 10.1155/grp/1205998

**Published:** 2026-09-06

**Authors:** Sapol Thepwiwatjit, Piyaporn Apisarnthanarak, Supot Pongprasobchai

**Affiliations:** ^1^ Division of Gastroenterology, Department of Medicine, Faculty of Medicine Siriraj Hospital, Mahidol University, Bangkok, Thailand, mahidol.ac.th; ^2^ Department of Radiology, Faculty of Medicine Siriraj Hospital, Faculty of Medicine Siriraj Hospital, Mahidol University, Bangkok, Thailand, mahidol.ac.th

**Keywords:** dyspepsia, esophagogastroduodenoscopy, transabdominal, ultrasound, uninvestigated dyspepsia

## Abstract

**Background and Aim:**

Dyspepsia is a common issue worldwide, primarily caused by gastroduodenal disorders. Esophagogastroduodenoscopy (EGD) is the standard investigation of uninvestigated dyspepsia (UID), whereas transabdominal ultrasonography (TUS) is used to evaluate various hepatopancreatobiliary diseases that may mimic dyspeptic symptoms. TUS is widely used among physicians, even though the diagnostic yield remains unclear. This study is aimed at assessing the additional benefit of TUS on EGD in investigating the etiologies of dyspepsia.

**Methods:**

This prospective study included patients with UID indicating for EGD at the Division of Gastroenterology, Department of Medicine, Faculty of Medicine Siriraj Hospital, Mahidol University, Bangkok, Thailand. All patients underwent both EGD and TUS within 1 month. Lesions identified by both methods were recorded and classified based on their potential to explain dyspepsia. Clinical data and reasons for EGD were also collected.

**Results:**

Three hundred and seventy patients with a mean age of 59 were included in the analysis. Dyspepsia‐relevant endoscopic findings were present in 24.1% with common diagnoses including erosive gastritis/duodenitis, peptic ulcer, and erosive esophagitis. *Helicobacter pylori* was detected in 23.5%. No dyspepsia‐relevant ultrasonographic findings were noted, though 9.5% of patients had ultrasonographic lesions requiring further investigation. No gastroesophageal and hepatopancreatobiliary cancers were detected.

**Conclusion:**

EGD provides some diagnostic information for dyspeptic patients, with additional TUS offering limited benefit. TUS findings rarely contributed to the diagnosis or necessitated further intervention, indicating that it does not enhance the diagnostic yield of EGD in this study.

## 1. Introduction

Dyspepsia is one of the common gastrointestinal (GI) disorders, affecting 20%–25% of population worldwide [1, 2]. Dyspepsia is defined as pain or discomfort in the upper abdomen and may include other symptoms such as epigastric pain or burning, sensation of fullness, early satiation, anorexia, belching, nausea and vomiting, upper abdominal bloating, and heartburn and regurgitation [3]. The etiologies of dyspepsia include atypical presentation of gastroesophageal reflux disease (GERD), peptic ulcer disease (PUD), erosive gastritis/duodenitis, symptomatic biliary stones, chronic pancreatitis, GI cancers (e.g., esophageal, gastric, hepatobiliary, and pancreatic), and functional dyspepsia [4–6]. Diagnosing dyspepsia based solely on upper abdominal complaints is challenging, as clinical symptoms often do not correlate well with underlying causes [7].

Uninvestigated dyspepsia (UID) refers to individuals with dyspeptic symptoms who have not undergone specific investigations. Both Thai and global guidelines recommend esophagogastroduodenoscopy (EGD) as the appropriate investigation for dyspeptic patients, particularly those age > 50 years old or with alarming symptoms. The Thai Dyspepsia Guideline 2018 recommends EGD for patients over 50 years old, or with clues of upper GI bleeding, early satiety, unexplained weight loss, persistent vomiting, a family history of upper GI cancer, or unresolved symptoms after a trial of proton pump inhibitors (PPI) for 4–8 weeks [3, 8]. However, EGD only diagnoses intraluminal pathologies. Other diagnostic tools, such as transabdominal ultrasonography (TUS), computed tomography (CT), magnetic resonance imaging (MRI), or endoscopic ultrasound (EUS), may help diagnose biliary, pancreatic, or liver diseases, with diagnostic yields of up to 64% in patients without organic causes detected by EGD [5, 9–11].

Hepatobiliary diseases are uncommon causes of dyspepsia [3, 8, 12], but the incidence of hepatocellular carcinoma and cholangiocarcinoma is higher in Thailand due to the high prevalence of hepatitis B and unhealthy eating habits, such as consuming raw fish [13, 14]. Pancreatic cancer may have very low pretest probability in patients presenting with dyspeptic symptoms; nevertheless, pancreatic cancer is one of the greatest concerns among doctors and patients. Current recommendations emphasize EGD for older patients and those with alarming symptoms or symptoms that remain unresolved following a trial of PPI, but there is no strong recommendation regarding the use of TUS in dyspeptic patients [3, 8, 15].

The proportion of dyspepsia etiologies identified by TUS in previous studies ranges from 5% to 45% [11, 16, 17]. This variation likely reflects differences in study inclusion criteria and the definitions used for ultrasonographic diagnoses. Despite most guidelines excluding TUS from the recommended diagnostic tools for dyspepsia, it remains a commonly used investigation among general practitioners, internists, surgeons, and gastroenterologists. This widespread use is often driven by clinical anxiety to rule out occult hepatopancreatobiliary malignancies and the high accessibility of the modality, even though its diagnostic utility and cost‐effectiveness in dyspeptic patients remain unclear.

This study is aimed at evaluating the diagnostic role of EGD and TUS in identifying the etiologies of dyspepsia.

## 2. Materials and Methods

### 2.1. Study Population

All Thai outpatients with UID indicating for EGD according to Thailand Dyspepsia Guideline 2018, who were referred to the Division of Gastroenterology, Faculty of Medicine Siriraj Hospital, Mahidol University, Bangkok, Thailand, between June 2023 and October 2024, were prospectively enrolled into the present study.

UID indicating for EGD is defined as patients with dyspeptic symptoms lasting for at least 4 weeks and presenting with one of the followings: (1) age of onset > 50 years, (2) evidence of upper GI bleeding, (3) early satiety, (4) unexplained weight loss, (5) persistent vomiting, (6) family history of upper GI cancer in a first‐degree relative, and (7) not resolved with a 4‐week trial of PPI.

Patients with obvious anemia, jaundice, and hepatomegaly upon physical examination; a prior diagnosis of upper GI cancer (including esophageal, gastric, small bowel, liver, pancreatic, and biliary cancer) or chronic pancreatitis; prior abdominal imaging within the last 6 months (including TUS, CT, or MRI); age < 18 years; or inability to provide informed consent for enrolment were excluded.

The sample size was calculated using the infinite population proportion formula, based on a 95% confidence interval (*Z* = 1.96) and an allowable precision error of 5% (*d* = 0.05). Drawing from previous literature where upper endoscopies (EGD) alone identified a diagnostic cause in 5%–45% of dyspeptic patients [11, 16, 17], combined with historical local data from Siriraj Hospital showing a baseline EGD yield of approximately 25% [6], the investigators clinically anticipated that adding routine TUS would optimize the cumulative diagnostic yield to 35% (*p* = 0.35). Applying these parameters yielded a baseline requirement of 350 participants, which was expanded by adding a 5% dropout buffer (requiring approximately 368.4 patients) and rounded up to establish a finalized target sample size of 370 patients.

**Figure 1:**
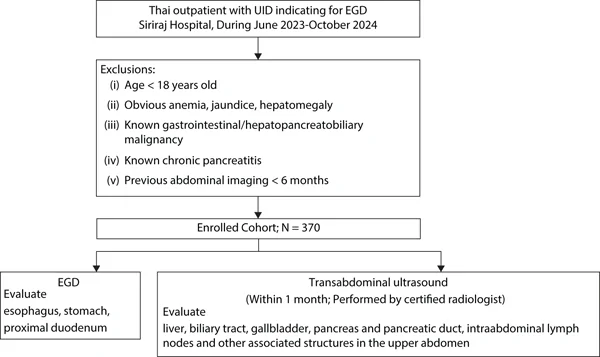
The flow diagram of study. EGD = esophagogastroduodenoscopy, UID = uninvestigated dyspepsia.

Based on these criteria and the sample size, 370 consecutive patients were enrolled into the present study. Patients were advised to undergo both EGD and TUS, and informed consent was obtained from participants. The flow diagram of study is demonstrated in Figure 1.

### 2.2. Data Collection

#### 2.2.1. Clinical Data

Before scheduling the EGD and TUS appointment, patients were clinically evaluated by the investigator at the time of presentation. A detailed structured questionnaire was administered, which included the following data: age, sex, unhealthy behaviors (such as smoking and alcohol consumption), family history of GI cancers, indications for EGD (including age of onset, alarm features, and failure to resolve with a PPI trial), use of aspirin and nonsteroidal anti‐inflammatory drugs (NSAIDs), and comorbidities.

#### 2.2.2. Endoscopic Data

EGD was performed to evaluate esophagus, stomach, and proximal duodenum. Endoscopic findings included GI malignancy, erosive esophagitis, esophageal ulcer, peptic ulcer, erosive gastritis/duodenitis, nonerosive gastritis/duodenitis, and gastroduodenal polyps.

Endoscopic findings classified as organic dyspepsia were peptic ulcer (gastric and duodenal), esophagitis, neoplasm, and erosive gastritis/duodenitis [18].

#### 2.2.3. Ultrasonographic Data

TUS was performed by a diagnostic radiologist to assess the liver, biliary tract, gallbladder, pancreas and pancreatic duct, intraabdominal lymph nodes, and other associated structures in the upper abdomen. TUS finding included liver mass, liver nodule, liver cyst, fatty liver, biliary dilatation, gallstone, gallbladder polyp, pancreatic mass, pancreatic cyst, pancreatic duct dilatation, presence of intraabdominal lymph nodes, and kidney lesion.

#### 2.2.4. Statistical Analysis

All statistical analyses were performed using the Statistical Package for the Social Science (SPSS 29.0 for windows: IMB Corp., Armonk, New York, United States). Descriptive statistics were used as appropriate.

The present study was approved by the Institution Review Board of the Faculty of Medicine Siriraj Hospital.

## 3. Results

There were 370 patients included in the present study.

### 3.1. Patient Characteristics

The baseline characteristics of the 370 patients and the indications for EGD in UID are shown in Table 1. Approximately three‐quarters of the patients were female, with a mean age of 59 years. Seventy‐six percent of them had at least one comorbidity. About 10%–15% of the patients were using ulcerogenic drug, whereas 76% of the patients had previously taken PPI.

**Table 1:** Baseline characteristics of 370 patients.

Characteristics	Result
Age, mean ± SD (years)	58.8 ± 11.9
Gender, female:male	289:81
Family history of upper GI cancer, *N* (%)	7 (1.9)
Comorbidities, *N* (%)	
Diabetes mellitus	55 (14.9)
Hypertension	155 (41.9)
Dyslipidemia	174 (47.0)
Cardiovascular diseases	24 (6.5)
Cirrhosis	2 (0.5)
Chronic kidney disease stage 3	14 (3.7)
Gynecologic disorder	20 (5.4)
Any	281 (75.9)
Medications, *N* (%)	
NSAIDs	56 (15.1)
Antiplatelets	36 (9.7)
Anticoagulants	5 (1.4)
Proton pump inhibitors	279 (75.4)
Unhealthy lifestyle, *N* (%)	
Cigarette smoking	61 (16.5)
Alcohol drinking	83 (22.4)
Duration of symptoms, median, IQR (months)	8, 5‐12
Indications for EGD, *N* (%)	
Age of onset > 50 years old	299 (80.8)
Presence of alarm symptoms	89 (24.1)
Not resolved with a trial of PPI	275 (74.3)

Abbreviations: EGD = esophagogastroduodenoscopy, GI = gastrointestinal, IQR = interquartile range, NSAIDs = nonsteroidal anti‐inflammatory drugs, PPI = proton pump inhibitor, SD = standard deviation.

### 3.2. EGD Findings

EGD revealed normal finding in 40 patients (10.8%) and at least one abnormal finding in 330 patients (89.2%). Erosive esophagitis was identified in eight patients (2.2%), of which seven were classified as Los Angeles (LA) grade A and one as LA grade C. Peptic ulcers were found in 12 patients (3.2%). Erosive gastritis/duodenitis was detected in 70 patients (18.9%), whereas nonerosive gastritis/duodenitis was observed in 234 patients (63.2%). Gastroduodenal polyps were found in 30 patients (9.1%).

*Helicobacter pylori* infection was identified in 87 patients (23.5%).

### 3.3. TUS Findings

TUS showed normal finding in 96 patients (25.9%) and at least one positive ultrasonographic finding in 274 patients (74.1%). All positive findings are listed in Table 2.

**Table 2:** Summary of findings on EGD and TUS in 370 patients.

Findings	*N* (%)
EGD	
Normal	40 (10.8)
Erosive esophagitis	8 (2.2)
Peptic ulcer	12 (3.2)
Erosive gastritis/duodenitis	70 (18.9)
Nonerosive gastritis/duodenitis	234 (63.2)
Gastroduodenal polyps	30 (9.1)
*Helicobacter pylori* infection	87 (23.5)
TUS	
Normal	96 (25.9)
Benign focal lesion in the liver	47 (12.7)
Hemangioma	6 (1.6)
Cyst	41 (11.1)
Inconclusive focal liver lesion	21 (5.6)
Signs of cirrhosis	3 (0.8)
Fatty liver	175 (47.3)
Pancreatic cystic lesion	2 (0.5)
Surgically absent gallbladder	24 (6.5)
Gallstones	39 (10.5)
Gallbladder polyp	30 (8.1)
Focal adenomyomatosis	13 (3.5)
Dilated common bile duct	9 (2.4)
Benign kidney lesions	79 (21.4)

Fatty liver was the most frequent ultrasonographic diagnosis (47.3%). Normal TUS was found in 25.9% of the patients. All inconclusive liver lesions were liver nodules of varying in sizes (ranging from tiny to 3.3 cm). Additional cross‐sectional imaging by CT or MRI, or interval follow‐up with TUS, confirmed that all of these lesions were nonspecific liver nodules, focal fat sparing, or benign focal lesions of the liver. No malignant liver lesions were identified. Two pancreatic cystic lesions (measuring 0.8 and 1.0 cm) were found, and further investigations by EUS or magnetic resonance cholangiopancreatography (MRCP) eventually revealed branch‐duct intraductal papillary mucinous neoplasm (IPMN). All gallbladder polyps were small (< 1 cm). Nine patients had common bile duct (CBD) dilatation. After further investigation, eight of these patients were found to have insignificant findings. However, one patient had a CBD stone from EUS. She underwent endoscopic retrograde cholangiopancreatography (ERCP) for stone removal, resulting in the resolution of her dyspeptic symptoms.

Thirty‐nine patients had gallstone disease. One of these 39 patients had additional signs of gallbladder wall thickening and gallbladder distension. This patient underwent laparoscopic cholecystectomy, and the pathological diagnosis of chronic cholecystitis was confirmed. After the procedure, this patient was free of dyspeptic symptoms.

### 3.4. Dyspepsia‐Relevant Findings From EGD and TUS

Dyspepsia‐relevant endoscopic findings, which were classified as organic dyspepsia, were found in 89 patients (24.1%). The remaining 281 patients (75.9%) had either normal or nondyspepsia‐relevant endoscopic findings.

On TUS, none of the positive findings were directly clinically relevant, and they were not categorized as true‐ or false‐positive findings. Thirty‐five patients (9.5%) had clinically actionable findings that required further investigation or treatment, such as chronic cholecystitis or CBD stone. However, the majority of patients (90.5%) had either normal ultrasonographic findings or findings that were neither related to dyspepsia nor clinically actionable.

The proportions of dyspepsia‐relevant findings are shown in Table 3.

**Table 3:** Dyspepsia‐relevant findings of the 370 patients.

Findings	*N*(%)
By esophagogastroduodenoscopy	
Dyspepsia‐relevant findings	89 (24.1)
Not relevant to dyspepsia	281 (75.9)
By transabdominal ultrasonography	
Dyspepsia‐relevant findings	0 (0)
Not relevant to dyspepsia, but clinically actionable	35 (9.5)
Not relevant to dyspepsia	335 (90.5)

## 4. Discussion

Dyspepsia is a challenging clinical issue in gastroenterology, particularly in terms of how and when to investigate dyspeptic patients. Moreover, identifying the causes of dyspepsia is complicated due to the lack of a global definition. This study presents the results from two common diagnostic investigations for dyspeptic patients.

According to Thailand′s dyspepsia guidelines, EGD is recommended for patients with UID when (a) age > 50 years, (b) the presence of alarm symptoms (evidence of upper GI bleeding, early satiety, significant weight loss, persistent vomiting, or family history of upper GI cancer), or (c) failure to respond to a trial of PPI [3]. These guidelines are in line with those from the Japanese Society of Gastroenterology and the British Society of Gastroenterology [19, 20]. In contrast, the American College of Gastroenterology and the Canadian Association of Gastroenterology (ACG‐CAG) clinical guideline recommends routine endoscopy only for patients over 60 years of age, with the alarm symptoms considered on an individual basis [8]. The results from the present study show that EGD had a diagnostic yield of 24.1%, which is lower than the 20%–50% reported in previous studies [18]. The proportion of dyspepsia‐relevant endoscopic findings was consistent with the results of the largest retrospective cohort from our center [6]. Erosive esophagitis accounts for 8%–10%, erosive gastritis/duodenitis in 15%–20%, and peptic ulcer in 3%–5%, consistent with finding from southern Thailand [6, 17].

Our results, along with the prior data from Thailand, show that EGD has a diagnostic yield of 24.1% in patients with UID. However, 2.2% of patients in our study had erosive esophagitis, with seven patients classified as LA grade A and one as LA grade C. LA grade A esophagitis can be found in 5%–7.5% in healthy individuals [21], making its role as the primary cause of dyspepsia controversial. No gastroesophageal cancer was detected, likely due to the low prevalence of upper GI cancer in Thailand [6], which may not be applicable in countries with a higher incidence of such cancers.

*H. pylori* infection was present in 23.5% of patients, consistent with our previous cohort [6]. *H. pylori* can cause erosive gastritis/duodenitis, and the benefit of EGD compared to *H. pylori* testing in younger patients remains an area for future investigation.

Regarding ultrasound, most guidelines do not support its routine use in investigating UID [3, 8, 20]. However, the Japanese Society of Gastroenterology suggests abdominal ultrasound for dyspeptic patients with alarm symptoms [19]. Our findings do not support routine ultrasound use, as no ultrasonographic findings were directly related to dyspepsia. Although an individual TUS is relatively inexpensive, its routine deployment across millions of dyspeptic patients worldwide creates a massive aggregate healthcare burden. Moreover, incidental findings such as small liver lesions, fatty liver, pancreatic cysts, and benign gallbladder lesions led to unnecessary subsequent investigations (such as CT or MRI), further increasing healthcare costs. Notably, no HPB cancers were detected in our cohort. The median symptom duration in our study was 8 months, which likely suggests benign diseases. Ultrasound may have higher utility in cases with a shorter symptom duration.

In previous studies, gallstone disease accounted for 20% of dyspeptic patients undergoing abdominal ultrasonography [11, 17, 22]. Our study found gallstones in 11% of patients, but the correlation between gallstones and dyspeptic symptoms remains debated [23]. Given conflicting data, we do not classify gallstone disease as a direct cause of UID, although one patient underwent laparoscopic cholecystectomy after ultrasound revealed additional findings, including gallbladder wall thickening and distension.

The present study was conducted at a large tertiary center in Thailand. The lack of direct ultrasonographic diagnoses and the low prevalence of dyspepsia‐relevant endoscopic findings in this study may be due to the prolonged symptom duration (median 8 months) and prior PPI use, which could mask significant findings.

Based on our findings, EGD should be considered for identifying etiologies in UID patients, whereas *H. pylori* testing may also be useful. Conversely, the routine use of TUS is not recommended; instead, it should be deployed selectively based on distinct clinical phenotypes. For instance, TUS serves as a critical diagnostic adjunct for patients with uncharacterized upper abdominal pain such as postprandial right upper quadrant tenderness or radiation to the back—even in the absence of classic alarm features like jaundice or palpable hepatomegaly. Furthermore, patients presenting with acute or subacute symptoms of short duration benefit significantly from targeted ultrasound, as sudden‐onset upper abdominal pain is more likely to stem from hepatobiliary pathologies, which EGD cannot detect.

The strengths of this study include (a) it being the first prospective study from Thailand evaluating both EGD and TUS in UID patients, (b) all TUS studies being conducted by certified diagnostic radiologists, and (c) the valuable clinical insights provided for UID diagnostic methods. Limitations include (a) the prolonged median symptom duration and low prevalence of upper GI cancer, leading to a higher likelihood of benign diagnoses, and (b) potential selection bias, as most patients had already received empirical PPI treatment before the investigation. This prior therapy could mask or heal active acid‐related intraluminal pathologies, potentially underestimating the true baseline diagnostic yield of EGD.

## 5. Conclusion

The diagnostic yield of EGD in UID may be useful for identifying the etiologies of dyspepsia. TUS detected no dyspepsia‐relevant lesions but revealed a few clinically actionable findings requiring further investigation or treatment. Additional TUS did not enhance the diagnostic yield of EGD and should not be routinely used in all UID patients, though it may be considered on a case‐by‐case basis.

## Funding

This study was supported by the Siriraj Research Development Fund (managed by Routine to Research: R2R), R016735006, the Division of Gastroenterology of the Faculty of Medicine Siriraj Hospital, and the Gastroenterological Association of Thailand.

## Disclosure

This work was previously presented as an oral presentation at the Asia Pacific Digestive Week (APDW) 2024 and published as an abstract in the *Journal of Gastroenterology and Hepatology.* The abstract is available at https://onlinelibrary.wiley.com/doi/10.1111/jgh.16779. All authors approved the submitted version. The study was reviewed and approved by the Siriraj Institutional Review Board. All study participants, or their legal guardian, provided informed written consent before study enrollment.

## Conflicts of Interest

The authors declare no conflicts of interest.

## Data Availability

The data that support the findings of this study are available from the corresponding author upon reasonable request.
